# Ramadan diurnal intermittent fasting is associated with significant plasma metabolomics changes in subjects with overweight and obesity: A prospective cohort study

**DOI:** 10.3389/fnut.2022.1008730

**Published:** 2023-01-09

**Authors:** Mohamed Madkour, Alexander D. Giddey, Nelson C. Soares, Mohammad H. Semreen, Yasser Bustanji, Falak Zeb, Rabih Halwani, MoezAlIslam E. Faris

**Affiliations:** ^1^Department of Medical Laboratory Sciences, College of Health Sciences, University of Sharjah, Sharjah, United Arab Emirates; ^2^Research Institute of Medical and Health Sciences (RIMHS), University of Sharjah, Sharjah, United Arab Emirates; ^3^Department of Medicinal Chemistry, College of Pharmacy, University of Sharjah, Sharjah, United Arab Emirates; ^4^Department of Human Genetics, National Institute of Health Doutor Ricardo Jorge (INSA), Lisbon, Portugal; ^5^Department of Basic Medical Sciences, College of Medicine, University of Sharjah, Sharjah, United Arab Emirates; ^6^School of Pharmacy, Department of Biopharmaceutics and Clinical Pharmacy, The University of Jordan, Amman, Jordan; ^7^Department of Clinical Sciences, College of Medicine, University of Sharjah, Sharjah, United Arab Emirates; ^8^Department of Clinical Nutrition and Dietetics, College of Health Sciences, University of Sharjah, Sharjah, United Arab Emirates

**Keywords:** omics, time-restricted eating, caloric restriction, intermittent fasting, metabolism

## Abstract

**Introduction:**

During the holy month of Ramadan, adult healthy Muslims are mandated to abstain from dawn to sunset, with free eating at night hours that may extend up to 12 h. The current work was designed to investigate the metabolomics changes incurred upon the observance of Ramadan diurnal intermittent fasting (RDIF).

**Methods:**

Twenty-five metabolically healthy participants with overweight and obesity (7 females and 18 males, with a mean age of 39.48 ± 10.0 years) were recruited for the study and were followed before and at the end of RDIF month. Dietary, anthropometric, biochemical, and physical activity assessments were performed before and at the end of the fasting month. The metabolomic assay was performed using liquid chromatography-mass spectrometry for the two-time points.

**Results and discussion:**

Metabolomics assay revealed a significant reduction in a few metabolites. The analysis revealed that 27 metabolites differed significantly (*P* < 0.05) between pre-and post-RDIF. Among the differentially abundant metabolites, 23 showed a decrease with fasting, these included several amino acids such as aspartame, tryptophan, phenylalanine, histidine, and other metabolites including valeric acid, and cortisol. On the other hand, only four metabolites showed increased levels after RDIF including traumatic acid, 2-pyrrolidinone, PC[18:1(9Z)/18:1(9Z)], and *L-sorbose*. The MetaboAnalyst^®^ platform reported that the top enriched metabolic pathways included: (1) histidine metabolism; (2) folate biosynthesis (3) phenylalanine, tyrosine, and tryptophan biosynthesis; (4) aminoacyltRNA biosynthesis; (5) caffeine metabolism; (6) vitamin B_6_ metabolism; and several other pathways relating to lipid metabolisms such as arachidonic acid metabolism, glycerophospholipid metabolism, and linoleic acid metabolism. In conclusion, RDIF entails significant changes in various metabolic pathways that reflect different dietary and lifestyle behaviors practiced during the fasting month.

## Introduction

Fasting, the abstinence from eating or drinking for a specific time, is one of the most commonly practiced dietary modifications by humans since ancient times, driven by various spiritual, religious, and health factors (1). Recently, growing attention has been directed toward intermittent fasting (IF)–an eating pattern that includes periods without food or with very little food–as a cost-effective, health-improving, and disease-preventing regimen. Extensively detailed reviews published recently unraveled that IF has a wide spectrum of positive effects against aging, and chronic cardiovascular, metabolic, and neurodegenerative diseases. Hence, IF continues to gain attention as a therapeutic and preventative intervention to counteract such chronic diseases (2–4). Given the radical changes in eating habits and lifestyle behaviors brought about by observing IF, a wide range of physiological effects are brought about, including unique metabolic shifts (4, 5).

Metabolomics is an advanced analytical technique to measure the quantitative abundance of large numbers of metabolites for a given sample type, that can map the metabolic profiles of human blood, for instance, and provide vital information related to the *in vivo* physiological states of the human body. Such states are affected by dietary, lifestyle, epigenetic, genetic, and physiological factors; thus, measuring the metabolome can enable a better understanding of the hidden metabolic relationships between these factors. Moreover, metabolomics allows the comprehensive evaluation of the metabolic mechanisms of physiological responses and diseases, as well as the biological effects of drugs, nutrients, and environmental influences (6).

Ramadan fasting, a mandatory obligation on adult Muslims observed during the ninth month of the lunar calendar, is one of the most commonly practiced forms of IF (5), with more than 1.5 billion followers throughout the globe, and considered one of the most extensively studied forms of religious fasting in the current era. Owing to its unique pattern of diurnal fasting for 29–30 consecutive days, involving complete abstinence from all food and drink, including water, from dawn to sunset (7); the Ramadan model of IF has previously been shown to yield a plethora of beneficial anthropometric, biochemical, and metabolic health effects. These include improved body anthropometrics (8–11), improved metabolic syndrome components (9), alleviating oxidative stress and inflammatory markers (12, 13), normalizing glucose homeostasis (14), and liver function markers (15), and reduced cardiometabolic risk factors (16) in healthy subjects. In a recent report by Mindikoglu et al. (17, 18) using proteomic analysis, the dawn-to-sunset model of IF for thirty consecutive days showed distinctive fingerprint modifications and revealed that this model of IF is associated with an anticancer proteomic signature, up-regulates key regulatory proteins of the immune system, lipid and glucose metabolism, DNA repair, circadian clock, cognitive function, and cytoskeleton remodeling in healthy people (17). Such proteomic changes reported during Ramadan are inevitably reflected in the metabolic mapping in the human body, which requires further investigation using advanced metabolomic techniques.

Considering the significant dietary and lifestyle changes associated with the observance of Ramadan diurnal intermittent fasting (RDIF), including macronutrients, food groups, (19), sleep duration and quality (20, 21), and physical activity (22) in comparison with the pre-and post-fasting days; the current study was designed to investigate the metabolomics changes associated with the observance of RDIF in metabolically healthy subjects with overweight and obesity. We hypothesized that Ramadan fasting month will be accompanied by distinctive metabolomic signatures and biochemical fingerprints that characterize it from non-fasting days.

## Materials and methods

### Subjects

This observational study included a convenience sample of overweight or obese adults from the United Arab Emirates (UAE), including expatriates from Jordan, Palestine, Syria, Egypt, and Sudan who were residing in Sharjah. Potential participants were invited to enroll in this study through social media, the hospital bulletin, and personal communications. Announcements containing the inclusion criteria were disseminated *via* social media and personal contacts. Those who were interested in the study contacted us and a formal meeting was arranged to discuss further the study objectives and protocol, sign the informed consent, and start the pre-fasting baseline measurements. All the study participants completed the study to the end, with 100% compliance with the study requirements. This was driven by participants’ enthusiasm to know how the observance of Ramadan fasting affects their body weight, metabolism, and other health measurements. Those who expressed willingness to participate were asked to visit the Sharjah University Hospital for a meeting.

Inclusion criteria were: healthy female or male adults (aged 18–60 years) who had not previously been diagnosed with any chronic or metabolic disease. Those with a history of cardiovascular or endocrine disease or diabetes mellitus were excluded along with pregnant women. Those who took medication 1 week before Ramadan, had bariatric surgery in the last 6–9 months or had engaged in a weight-reducing regimen 1 month before the commencement of Ramadan were also excluded. The daily fasting duration in this study was approximately 15 h. Only overweight or obese [body mass index (BMI) ≥ 25 kg/m^2^] adult female or male Muslims who expressed their willingness to fast Ramadan were included. The University of Sharjah Research and Ethics Committee (REC-16-05-11-01) approved this study. All participants signed a consent form before being enrolled in this study.

### Study design

Our prospective study was executed during Ramadan (from June 2016 to July 2016, corresponding to Ramadan month in the 1438 *Hijri* of the lunar calendar), where the daily fasting period covered nearly 15 h. Data were collected 1 week before Ramadan (pre-fasting or baseline) and after completing 28–30 days of Ramadan (at the end of Ramadan). During Ramadan, fasting people abstain from food and drink (including water) and do not smoke from dawn to sunset. We compared the studied variables for each participant before and during Ramadan, meaning each participant served as their control. Participants did not receive any nutritional recommendations or physical activity advice at any stage during this study. The Islamic laws of Ramadan excuse females from fasting during Ramadan while their menstrual period; therefore, the fasting days for female participants were about 23–25 days.

### Anthropometric assessment

Waist circumference (WC) (to the nearest 0.1 cm), body weight (to the nearest 0.1 kg), and height (to the nearest 0.1 cm) were measured using standardized techniques (23, 24). Height (without shoes) was measured using a portable stadiometer (Seca Gmbh and Co., Germany Model 217), while weight (without excess clothes) was measured using a body weight scale (Detecto, MO/USA). WC was measured at the umbilicus with participants in a standing position and breathing normally. BMI (kg/m^2^) was calculated accordingly and classified (25). Diastolic and systolic blood pressure (DBP and SBP) were measured with participants in a sitting position using a digital upper arm monitor (Omron Healthcare Inc., Japan Model BP742N).

### Biochemical assessment

Venipuncture blood sampling (10 ml) was undertaken for all participants 8–10 h after fasting (approximately between 11 a.m. and 1 p.m.). Within 3 h of collection, whole blood samples were centrifuged, separated, and the serum samples were frozen at −80°C. A fully automated clinical chemistry analyzer (Adaltis, Pchem1, Rome/Italy) was used to quantify fasting plasma glucose and serum lipid profiles (TC, LDL-C, HDL-C, and TG). Enzyme-linked immunosorbent assay (ELISA) kits (Elabscience, USA) were used to measure fasting insulin, adiponectin, and leptin. Serum levels of PCSK9 were also measured using ELISA kits (Aviscera Bioscience Inc., CA, USA). The homeostatic model assessment of insulin resistance (HOMA-IR) was calculated.

### Dietary intake assessment

Assessment of food intake was performed using the 24-hour food recall technique. Food intake information was collected for 3 days (two weekdays and one weekend day) before and during Ramadan by trained dieticians. Two-dimensional food models were used to help participants remember and approximate the food portion sizes consumed. Intakes of total calories, macro-, and micronutrients were assessed using Food Processor software (version 10.6 ESHA Research, Salem, OR/USA).

### Physical activity level

The Dietary Reference Intakes classification for general physical activity level was used to assess participants’ level of physical activity (26). This classification depends on the general physical exercise pattern. Participants were considered highly active if they performed at least 2 h/day of moderate-intensity physical exercise or 1 h of vigorous exercise in addition to daily living activities. Participants were considered moderately active if they performed more than 1 h/day of moderate-intensity exercise in addition to daily living activities. Participants that performed 30 min to 1 h/day of moderate-intensity physical exercise in addition to daily living activities were considered to have low activity. Finally, participants who performed daily living activities without other physical exercise were considered sedentary (26).

### Metabolomic assay using liquid chromatography mass spectrometry

#### Reagents

Formic acid and acetonitrile were obtained from Fisher Chemical (Loughborough, UK). Methanol (≥99.9%) was purchased from Honeywell (Germany). Deionized Water (LC-MS grade, CHROMASOLV) was purchased from Honeywell (Germany).

#### Sample collection and preparation

A total of 4 ml of blood was then collected from each subject into a sterile container. The samples were stored immediately at −80°C for long-term storage until further metabolomics analysis. For analysis, an aliquot of thawed plasma sample was transferred into a microcentrifuge tube and cold methanol was added at 3:1 v/v (i.e., 30 μl sample, add 90 μl cold methanol), vortexed, and allowed to sit at −20°C for 2 h. Next, samples were centrifuged at 20,817 × *g* for 15 min at 4°C and the supernatant was transferred to a new microcentrifuge tube. Usually, the transferred volume was three times that of the original sample (i.e., for a 30 μl sample, add 90 μl cold methanol, then transfer 90 μl supernatant). The samples were dried using a Speed vac at 30–40°C and the dried sample was stored in a −80°C freezer for further use or dissolved in a solvent for LC-MS/MS analysis. Samples were re-suspended in the starting solvent (0.1% formic acid) where the volume was three times the original plasma volume, i.e., 90 μl for 30 μl serum/plasma aliquot.

#### Liquid chromatography (Q-TOF MS)

TimsTOF Mass Spectrometer (BRUKER, Germany) along with Metaboscape software version 4 was employed for the detection and feature extraction/identification of the cell metabolites. Samples were chromatographically separated using a HAMILTON^®^ Intensity Solo 2 C_18_ column (100 um × 2.1 mm × 1.8 μm) and the Elute UHPLC Pump HPG 1,300 and autosampler and column oven. The elute autosampler temperature was set at 8°C and the column oven temperature was at 35°C. Metabolites were analyzed in auto MS/MS positive scan mode within the range of 20–1,300 m/z utilizing electrospray ionization (ESI). The ESI source was 10 L/min and the drying temperature was equal to 220°C.

The capillary voltage of the ESI was 4,500 V with 2.2 bar nebulizer pressure. The collision energy was set at 7 eV and the end Plate Offset at 500 V. Sodium Formate was used as a calibrate for the external calibration step and injected between 0 and 0.3 min in the gradient. For metabolite analysis, solvent A (Water + 0.1% FA) and solvent B (Acetonitrile + 0.1% FA) were used in gradient elution mode. The flow rate was set as (0.250–0.350 ml/min) for 30 min in gradient mode with a maximum pressure of 14,993 psi. And a total volume of 10 μl was injected into the QTOF MS. LC total ion chromatograms (TIC) and fragmentation patterns of the metabolites were identified using HMDB Mass Spectral Library (two technical replicates).

#### Data processing, analysis, and statistical approach

Processing and statistical analysis were performed using MetaboScape^®^ 4.0 software (Bruker Daltonics). Bucketing in T-ReX 2D/3D workflow the parameters set for molecular features detection were as follows- intensity threshold equal to 1,000 counts along with minimum peak length equal to seven spectra were fixed for peak detection using peak area as feature signal. The mass recalibration was done within the retention time range between 0 and 0.3 min. Representative MS/MS spectra import was set to be done by average. Parameters for data bucketing were assigned as follows: The retention time range started at 0.3 min and ended at 25 min, while the mass range started at 50 m/z and ended at 1,000 m/z.

#### Data and statistical analysis

The MetaboScape 4 quantitation values for technical duplicates of those identified metabolites were combined by taking the median of the two values (where both replicates reported a quantitative value), or else taking only the non-zero value (if the metabolite was not quantified in both replicates). Statistical analysis was performed utilizing independent, two-tailed Student’s *t*-tests and the *p*-values were adjusted for multiple testing corrections by the Benjamini-Hochberg method. The volcano plot was constructed using these adjusted *p*-values and log2-transformed fold changes (Post-Fasting/Pre-Fasting) and metabolites were labeled as significantly altered in abundance if they met both criteria of the adjusted *p*-value < 0.05 and the |log2(fold-change)| > 1. The heatmap was generated using the “pheatmap” package (1, 27) in R with row scaling applied. The principal components analysis was performed in R using “prcomp” from the “stats” package with scaling of variables applied. Functional Enrichments were constructed using MetaboAnalyst.

All data including the raw files have been deposited (or are available) to Metabolomics Workbench and the data track ID is provided. Other classical statistical analyses were performed using SPSS version 17.0 and reported based on the Strengthening the Reporting of Observational Studies in Epidemiology (STROBE) guidelines (28). Categorical variables were expressed as the frequency of occurrence and percentage (for sociodemographic data: sex, marital status, nationality, educational level, and physical activity level). Continuous variables were expressed as the mean ± standard deviation (SD) for normally distributed data of age, anthropometric, biochemical, and dietary data. Paired *t*-test were used to compare the changes in studied basic anthropometric, dietary and biochemical variables before and during fasting. All data were tested at a 5% level of significance (*P* < 0.05).

## Results

In total, 25 adult participants (7 females and 18 males) completed this observational prospective cohort study. Table 1 presents the participants’ sociodemographic characteristics. The mean age was 39.48 ± 10.0 years. Eight participants were obese and 17 were overweight. Before Ramadan fasting the SBP and DBP for all participants were 121.18 ± 10.46 and 71.57 ± 7.18 mmHg, respectively. The majority of participants held an undergraduate degree (72%), were of low physical activity (92%), and were from non-Gulf Cooperation Council (non-GCC) countries (88%).

**TABLE 1:** Participants’ sociodemographic characteristics (n = 25).

Characteristic		*n* (%)
**Age (Years)**		39.48 ± 10.0
**Sex**		
	Male Female	18 (72) 7 (28)
**Nationality**		
	UAE and other GCC countries	3 (12)
	Non-GCC	22(88)
**Marital status**		
	Married	20 (80)
	Single	5 (20)
**Educational level**		
	Basic education	3 (12)
	Undergraduate studies	18 (72)
	Postgraduate studies	4(16)
Body mass index (BMI, kg/m^2^)	Overweight (25–29.9)	17 (68)
	Obese (≥30)	8 (32)
Blood pressure (mmHg)*		
	Systolic blood pressure (mmHg)	121.18 ± 10.46
	Diastolic blood pressure (mmHg)	71.57 ± 7.18
**Physical activity**		
	Sedentary	2 (8)
	Low activity	23 (92)
	Moderately active	0
	Highly active	0

GCC, Gulf Corporation Council; UAE, the United Arab Emirates.

Significant reductions were reported in body weight, waist circumference, and LDL cholesterol, concomitant with significant increases in fasting blood glucose (still within normal ranges) and HDL cholesterol at the end of Ramadan fasting when compared with the pre-fasting levels. The dietary assessment revealed significant increases in PUFA, total carbohydrates, and total sugars, alongside a significant decrease in dietary cholesterol intakes during Ramadan in comparison with the pre-fasting levels (Table 2).

**TABLE 2:** Changes in anthropometric, biochemical and dietary intake variables before and during Ramadan fasting month.

Parameter	Before Ramadan	During Ramadan	*P-*value
Body mass index, BMI (kg/m^2^)	29.09 ± 4.76	28.53 ± 4.78	**0.0001**
Waist circumference, WC (cm)	96.34 ± 12.96	95.84 ± 12.86	**0.06**
Systolic blood pressure, SBP (mmHg)	121.18 ± 10.46	121.47 ± 9.96	0.435
Diastolic blood pressure, DBP (mmHg)	71.57 ± 7.18	70.2 ± 8.81	0.211
Fasting blood glucose, FBG (mg/dl)	99 ± 20.89	108.4 ± 20.47	**0.036**
Total cholesterol, TC (mg/dl)	167.3 ± 43.9	169.4 ± 34.7	0.295
High-density lipoprotein-cholesterol, HDL-C (mg/dl)	44.36 ± 7.31	58.97 ± 11.13	**0.0001**
Triglycerides, TG (mg/dl)	86.06 ± 50.13	89.36 ± 41.09	0.269
Low-density lipoprotein-cholesterol, LDL-C (mg/dl)	105.7 ± 36.35	92.63 ± 31.36	**0.0013**
**Dietary intake**			
Total calories (kcal/day)	2101.2 ± 570.3	2200.5 ± 882.4	0.235
Proteins (g/day)	105.2 ± 33.18	94.0 ± 49.9	0.119
Total fats (f/day)	71.17 ± 23.76	76.22 ± 37.00	0.192
Saturated fats (g/day)	21.43 ± 8.13	23.00 ± 11.9	0.221
MUFA (g/day)	19.78 ± 11.96	23.04 ± 13.98	0.148
PUFA (g/day)	8.84 ± 6.00	14.45 ± 13.7	**0.024**
Cholesterol (mg/day)	375.8 ± 145.2	261.4 ± 189.2	**0.016**
Total carbohydrates (g/day)	264.3 ± 89.04	292.86 ± 131.7	**0.071**
Total sugars (g/day)	68.56 ± 33.96	111.03 ± 61.58	**0.0001**

Bold numerals reflect significantly changed variables.

In this study, we examined a total of 50 plasma blood samples, 25 of which were collected pre-fasting and 25 of which were collected at the end of the fasting month (on days 29–30 of Ramadan). HPLC-MS/MS was used to analyse each plasma metabolite extract in duplicate. MS/MS spectra, retention time values, precursor m/z, and isotopic patterns were used to characterize identified compounds that were matched to Bruker’s implementation of the Human Metabolome Database (4.0). Where multiple features matched to a given database identification, only the feature with the highest Annotation Quality score (AQ score) was retained–i.e., only those features which best fit their respective database identifications concerning all the foregoing compound characteristics (isotopic pattern, precursor m/z, MS/MS spectra, retention time) were considered for further analysis. We retained 89 highly confidently assigned metabolites after filtration (see Supplementary Table 1).

**FIGURE 1:**
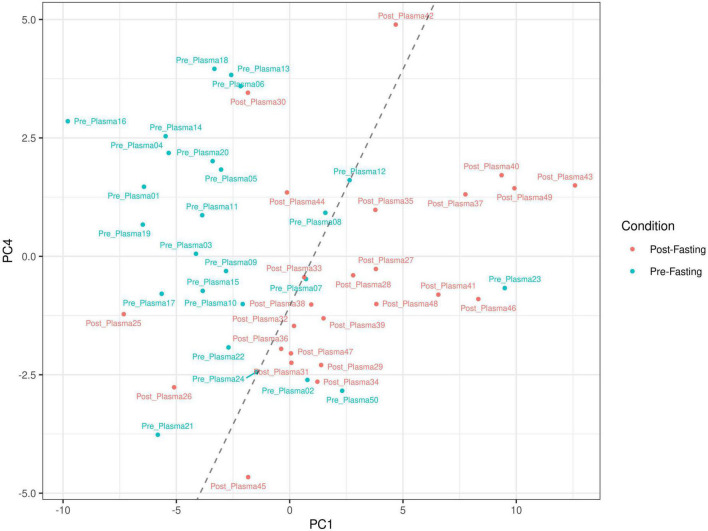
Principal component analysis based on the metabolic profile measured in 50 blood plasma samples before and after intermittent fasting. Plots show sample clustering with blue and red dots indicating samples pre- and post-fasting. The dashed line is a visual aid to assist in visualizing the separation between the groups along the first and fourth principal components (PC1 and PC4).

**FIGURE 2:**
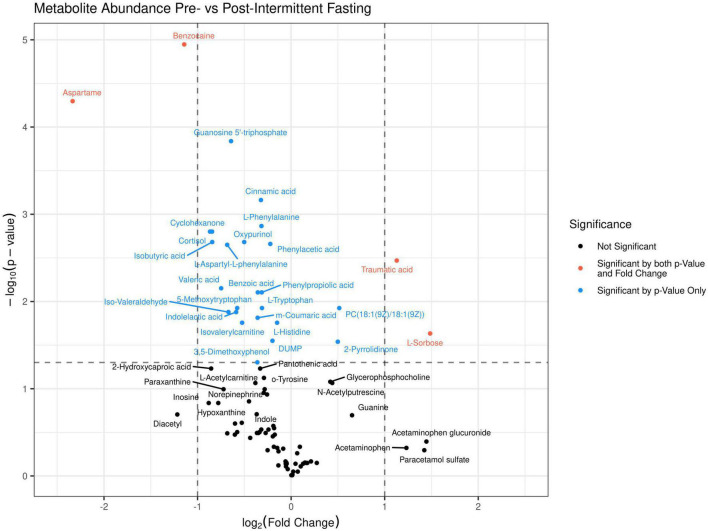
A volcano plot was generated for the 89 identified metabolites in the untargeted LC-MS/MS metabolomics analysis. Above the dashed horizontal line are those metabolites that significantly differed (*P* < 0.05) between before (dots to the left) and after fasting (dots to the right), in red are those metabolites that change by a Log2FC > 1, and in blue metabolites that change by a Log2FC < 1.

**FIGURE 3:**
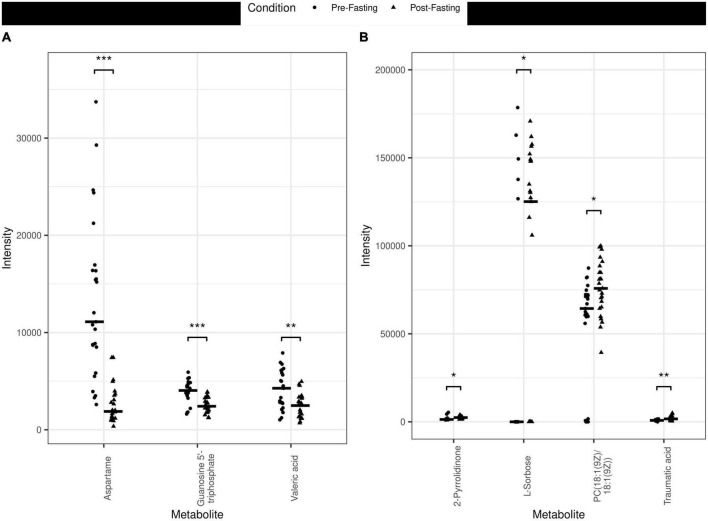
Jitter plots of the metabolites measured in the different plasma samples pre- vs. post-fasting. **(A)** Examples of metabolites that decreased with fasting are Aspartame (*P* < 0.001, ***), Guanosine 5′-triphosphate (*P* < 0.001, ***), and Valeric acid (*P* < 0.01, **). **(B)** Examples of metabolites that increased with fasting include 2-Pyrrolidinone (*P* < 0.05, *), L-Sorbose (*P* < 0.05, *), PC[18:1(9Z)/18:1(9Z)] (*P* < 0.05, *) and Traumatic acid (*P* < 0.01) (**).

The principal component analysis (PCA) of the metabolic profile of the measured samples revealed that samples mostly separated along the first principal component, PC1, and this separation was improved when considered in conjunction with PC4, enabling us to distinguish between the pre-and post-fasting groups (Figure 1, see also Figure 5). This prompted us to carry out a Student’s *t*-test to investigate the effect of fasting on each metabolite. The analysis revealed that 27 metabolites differed significantly (*P* < 0.05) between pre- and post-fasting. Among the differentially abundant metabolites, 23 showed a decrease with fasting (Figure 2 and Table 3), these included several amino acids such as aspartame, tryptophan, phenylalanine, histidine, and other metabolites including valeric acid, cortisol (see Supplementary Table 1 and Figure 3A). On the other hand, only four metabolites showed increased levels with fasting including compounds such as traumatic acid, 2-pyrrolidinone, PC[18:1(9Z)/18:1(9Z)], and L-Sorbose (see Table 4 and Figure 3B).

**FIGURE 4:**
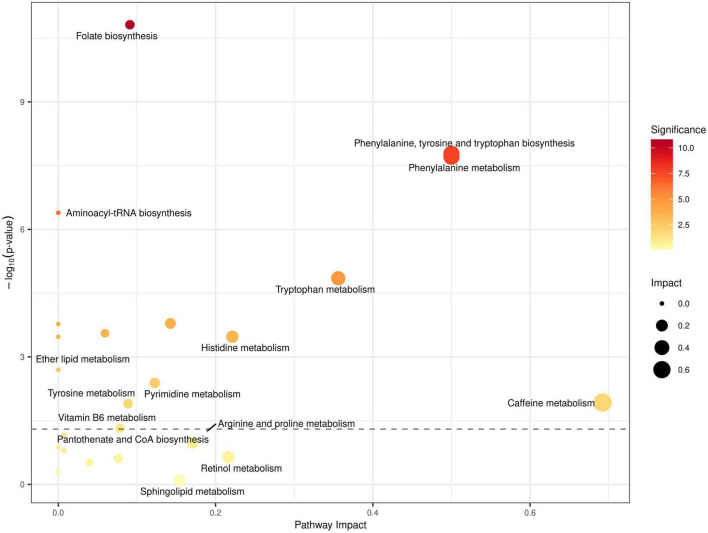
Quantitative metabolic pathway enrichment analysis generated *via* MetaboAnalyst^®^, considering altered abundance profiles with respect to fasting status for all identified metabolites. Points represent metabolic pathways, color gradient and circle size represent enrichment significance (yellow: higher *P*-values and red: lower *P*-values) and pathway impact score (larger for higher impact score), respectively. *x*- and *y*- axes represent the pathway to impact and the negative logarithm of significance (higher implies smaller *p*-values), respectively, with the dashed line representing the significance threshold cut-off of *P* = 0.05.

**FIGURE 5:**
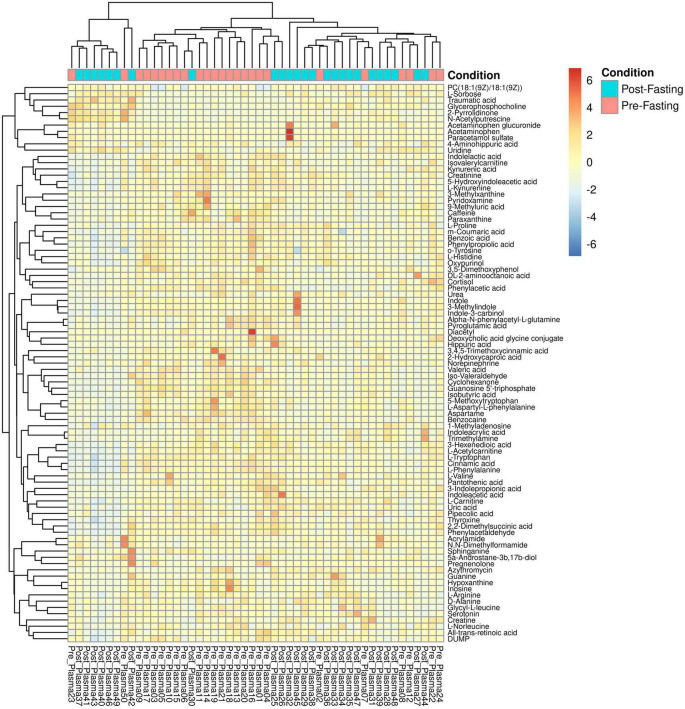
Heatmap and hierarchical clustering of the 89 identified metabolites and 50 samples. Columns represent samples, rows represent metabolites, and the relative abundance of the metabolites is displayed by color. The sample time point as either pre- (red) or post-fasting (blue) is indicated by the top-most annotation row.

**TABLE 3:** Summary of metabolites that decreased after Ramadan fasting.

Metabolite	HMDB ID #	p.log10	log2 fold change “pre”/“post”	HMDB link*
Aspartame	0000191	4.297	−2.33	https://hmdb.ca/metabolites/HMDB0000191
Cortisol	0000063	2.801	−1.143	https://hmdb.ca/metabolites/HMDB0000063
Isobutyric acid	0001873	2.681	−0.844	https://hmdb.ca/metabolites/HMDB0001873
Cyclohexanone	250654	2.800	−0.843	https://hmdb.ca/metabolites/HMDB0250654
Valeric acid	00892	2.152	−0.748	https://hmdb.ca/metabolites/HMDB0000892
L-Aspartyl-L-phenylalanine	0000706	2.649	−0.683	https://hmdb.ca/metabolites/HMDB0000706
Guanosine 5’-triphosphate	0001273	3.838	−0.641	https://hmdb.ca/metabolites/HMDB0001273
Iso-Valeraldehyde	0006478	1.879	−0.670	https://hmdb.ca/metabolites/HMDB0006478
Indolelactic acid	0000671	1.879	−0.587	https://hmdb.ca/metabolites/HMDB0000671
5-Methoxytryptophan	02339	1.924	−0.576	https://hmdb.ca/metabolites/HMDB0002339
Isovalerylcarnitine	00688	0.0001	1.431	https://hmdb.ca/metabolites/HMDB0000688
Oxypurinol	0000786	2.682	−0.501	https://hmdb.ca/metabolites/HMDB0000786
3,5-Dimethoxyphenol	0059966	1.302	−0.361	https://hmdb.ca/metabolites/HMDB0059966
Benzoic acid	0034158	0.0000	1.28	https://hmdb.ca/metabolites/HMDB0034158
m-Coumaric acid	0001713	1.813	−0.358	https://hmdb.ca/metabolites/HMDB0001713
Benzoic acid	0001870	2.104	−0.356	https://hmdb.ca/metabolites/HMDB0001870
Cinnamic acid	0000567	3.163	−0.323	https://hmdb.ca/metabolites/HMDB0000567
Phenylalanine	0000159	2.865	−0.317	https://hmdb.ca/metabolites/HMDB0000159
Phenylpropiolic acid	0002359	2.104	−0.314	https://hmdb.ca/metabolites/HMDB0002359
Tryptophan	0000929	1.924	−0.311	https://hmdb.ca/metabolites/HMDB0000929
Phenylacetic acid	0000209	2.660	−0.222	https://hmdb.ca/metabolites/HMDB0000209
dUMP	0001409	1.549	−0.199	https://hmdb.ca/metabolites/HMDB0001409
Histidine	0000177	1.756	−0.149	https://hmdb.ca/metabolites/HMDB0000177

*Source https://hmdb.ca/ version 5 2021, search filtered by biospecimen “blood”.

#Means “Number”.

**TABLE 4:** Summary of metabolites that increased after Ramadan fasting.

Metabolite	HMDB ID #	p.log10	log2 fold change “pre”/“post”	HMDB link*
PC[18:1(9Z)/18:1(9Z)]	0000593	0.0094	−1.147	https://hmdb.ca/metabolites/HMDB0000593
2-Pyrrolidinone	0002039	1.538	0.598	https://hmdb.ca/metabolites/HMDB0002039
Traumatic acid	0000933	2.469	1.129	https://hmdb.ca/metabolites/HMDB0000933
Sorbose	001266	1.633	1.486	https://hmdb.ca/metabolites/HMDB0001266

*Source https://hmdb.ca/ version 5 2021, search filtered by biospecimen “blood”.

#Means “Number”.

### Analysis of metabolic pathways

To better understand how RDIF affects metabolic pathways, we used the MetaboAnalyst platform to perform pathway enrichment and topological analysis on all identified and quantified metabolites. The MetaboAnalyst^®^ platform reported prominent metabolic pathways that showed enrichment for dysregulation based on the altered abundances of all identified metabolites in our data, as shown in Figure 4. The top enriched metabolic pathways included: (1) Histidine metabolism; (2) Folate biosynthesis (3) Phenylalanine, Tyrosine, and Tryptophan biosynthesis; (4) Aminoacyl-tRNA biosynthesis; (5) Caffeine metabolism (6) Vitamin B_6_ metabolism; and several other pathways relating to lipid metabolism such as Arachidonic acid metabolism, Glycerophospholipid metabolism, and linoleic acid metabolism (see Figure 4).

## Discussion

The significant reductions in body anthropometry and improvements in the lipid profile reported in the current work are consistent with the established improvements in body weight and cardiometabolic risk factors reported during Ramadan, as revealed by several previously published systematic reviews and meta-analyses (8, 9, 14, 16). The main finding of the current work involves that a great majority of altered metabolites decreased during fasting and only four increased significantly.

According to the available literature and the best knowledge of the authors, none of the previously published works had used non-targeted liquid chromatography-mass spectrometry (LCMS) in exploring the metabolomic changes upon Ramadan fasting in subjects with overweight/obesity. Such advanced technology is characterized by its power to detect large numbers of metabolites and intermediary compounds in human blood. The PCA analysis in Figure 1 clearly shows that fasting causes changes in metabolic plasma profiles, distinguishing samples from two populations before and after fasting, with few outliers on opposite sides e.g., Pre-fasting sample numbers 02, 07, 23, and 50 and post-fasting samples 25, 26, 30, 42, and 44 (see Figure 1). The main findings of the current work included significant increases in the blood levels of several metabolites that include lipids [PC(18:1(9Z)/18:1(9Z)], amides (2-pyrrolidinone), and Carboxylic acids (traumatic acid) (Table 4). However, a larger number of metabolites were decreased by the end of Ramadan fasting, including cortisol, aspartame, valeric acid, isobutyric acid, L-aspartyl-L-phenylalanine, and other metabolites (Table 3).

The lack of detection for the branched-chain amino acids (valine, leucine, and isoleucine) in the current work is consistent with what was mentioned earlier about the lack of chronic energy shortage during the fasting month. Considering the intermittent nature of Ramadan fasting that extends from 12–17 h, with the free eating pattern practiced during the night hours, along with the decreased physical activity and increased sedentary lifestyle during the day hours observed in many people, the release of BCAA becomes less demanded. This contradicts what was found upon prolonged fasting during 34–58 h fasting of four healthy volunteers, where a significant increase was detected in the blood levels of BCAA as a response to the chronic, stressful shortage in energy supply (6). It is well evident that BCAAs are mainly released from skeletal muscles, followed by uptake into the citric acid cycle for the energy metabolism pathway, or hepatic lipogenesis pathway after prolonged fasting or starvation (29).

Among the few studies that examined the metabolomic changes associated with Ramadan fasting is that of Mathew et al. (30). In their study, a targeted metabolomics approach was applied to blood samples of eleven healthy male volunteers, taken directly before and 2 h after consumption of a controlled meal in the evening at the beginning and the end of Ramadan, and after an overnight fast several weeks after Ramadan. The targeted metabolomics applied therein resulted in the detection and quantification of a total of 202 metabolites. These metabolites included amino acids, bile acids, acylcarnitines, and polyamines (30).

We observed that aspartame, an artificial sweetener, decreased after fasting by using non-targeted metabolomics. In contrast, previous studies revealed upon targeted and non-targeted metabolomics that sugar and its metabolites were increased after long- (58 h) and short-term fasting (15 h) confirming the expected physiological response to food intake (6, 30). These physiologically plausible responses were further attributed to an increase in bile acid and amino acid levels and a decrease in long-chain acyl-carnitine and polyamine levels. Intriguingly, we have identified a decrease in the fatty acid iso-butyrate, which is supported by a previous study that showed the concentrations of the number of phospholipids decreased after 26 days of fasting (30). In contrast, other studies demonstrated that short-chain fatty acids (SCFAs) like butyrates (2-hydroxybutyrate, 3-hydroxybutyrate, 2-keto-butyrate, and 2-amino-butyrate) were increased in the plasma after fasting (6, 31). The subjects in these studies, however, were lean, and hence the decrease in iso-butyrate we observed could be specific to overweight/obese subjects. Since SCFA are known to regulate the function of several innate immune cells as well as modulate antigen-specific immunity, lower levels of these fatty acids could help regulate the chronic low-grade inflammation associated with obesity. Although purines such as urate and uridine are known to increase upon fasting for the regulation of energy homeostasis (32, 33), additionally supported by another study which revealed that purines (xanthan, cytidine) were significantly increased in the plasma of four study volunteers (6), still in our study we observed a decrease in the level of purines such as oxypurinol. These discrepancies may be due to differences in sample size and fasting duration. Upon prolonged fasting, various metabolites were associated with the metabolic switching after glycogen storage depletion, including branched-chain amino acids (BCAAs), butyrates, and carnitines. More recently, other metabolites have been identified after prolonged fasting including increased levels of anti-oxidative metabolites and metabolites of the pentose phosphate pathway as well as transcriptional modulators such as signaling metabolites (3-hydroxybutyrate and 2-oxoglutarate) and purines/pyrimidines (29). However, such metabolomic exacerbations are not expected to be exhibited during Ramadan IF, during which *ad libitum* eating is allowed during the night hours, without restriction on any of the *Halal* foods, with an eating window of 7–12 h (corresponding to 12–17 h of daily fasting based on the solar season that crosses with the lunar month of Ramadan).

Cortisol is a glucocorticoid hormone and high levels thereof are associated strongly with stress-related disorders (34, 35). In this study, we showed that the hormone-like cortisol was significantly reduced after practicing 30 days of RDIF. Similarly, a recent study also demonstrated a significant decrease in the serum cortisol level after 30 days of DIRF in 34 healthy individuals (36). This will most likely be translated into better overall surveillance immunity. In addition, Vasaghi-Gharamaleki’s study also found that cortisol output and concentration significantly reduced after RDIF compared to baseline and this decrease lasted for 3 weeks after Ramadan (37). However, such a finding contradicts the lack of changes in salivary cortisol among Ramadan-fasting people (38).

Traumatic acid, a monounsaturated dicarboxylic acid, is originally a plant-derived “wound healing hormone.” However, this dicarboxylic acid metabolite could be detected in the human serum as part of the dicarboxylic acid derivatives that are increased upon ketogenesis. Furthermore, our study elucidated the positive effect of intermittent fasting on elevating levels of traumatic acid after RDIF. The previous study has revealed the antioxidant characteristics and collagen biosynthesis stimulating effect of traumatic acid, and it has been considered a potential agent in the treatment of many skin diseases (39, 40). Moreover, our study shows a significant increase in the rare monosaccharide sorbose, which is a poorly digestible sugar. So far, no human studies have investigated sorbose. However, many studies illustrate the effect of dietary intake of sorbose on animals. Sorbose feeding caused body weight loss in rats as described by Tamura et al. (41). It was also shown to ameliorate hyperglycemia, hyperinsulinemia, and hyperphagia in gold thio glucose (GTG)-injected obese mice [Kita et al. (42)]. Taking into consideration these findings will demonstrate the decisive impact of RDIF on the sugar metabolites.

Our study reveals a decreased level of L-phenylalanine which plays a main role in the biosynthesis of other amino acids. Despite the crucial role of phenylalanine in the synthesis of protein and many other molecules, the toxic effect and the neural cell damage caused by phenylalanine accumulation are well-identified (43). Our study discloses the potential positive impact of RDIF on the early prevention of cognitive decline by showing the reduced levels of phenylalanine after RDIF. These findings are supported by the observation of increased levels of phenylalanine metabolites in the non-fasting group of a Mild Cognitive Impairment in the elderly study when compared to the fasting group (44). Also, tyrosine and other aromatic chain amino acids were decreased after RDIF in the current study. Tyrosine metabolites are elevated in incident diabetes in south Asian individuals (45), therefore, the reduced level of this amino acid after RDIF attests to the diabetes-preventative characteristic of intermittent fasting.

Tryptophan and phenylalanine are essential for immune cells, especially T cell, activation. The observed decrease of these amino acids in the blood of overweight/obese subjects following fasting could help in manipulating the chronic low-grade inflammation associated with obesity. The current study reported an elevated level of glycerylphosphorylcholine (GPC) after the RDIF. While GPC was not itself significantly dysregulated (*P* < 0.1), glycerophospholipid metabolism, as a pathway, was significantly enriched for dysregulation (see Figure 4). GPC is a natural brain choline compound that influences Alzheimer’s disease and dementia treatment (46, 47). Also, GPC accumulation has a protective effect from the high interstitial concentrations of NaCl and urea in renal medullary cells during the renal concentrating mechanism (48). Moreover, GPC metabolites play a role in modulating the risk of cardiovascular disease *via* the atherosclerosis pathway (49). In summary, it is well documented that RDIF significantly affects the dietary intake, body composition, and blood profile of healthy and obese individuals. In essence, these changes may exert some changes in the metabolomics profile of obese and overweight individuals with the observance of RDIF. The relatively small sample size, the inability to generalize metabolomic changes associated with RIF to all fasting people due to different dietary and lifestyle behaviors, and the lack of causality due to the observational nature of the current work; are all among the limitations of this study.

## Conclusion

Our findings showed that RDIF is associated with a metabolomic signature that reflects the various dietary and lifestyle behaviors practiced during the day hours and the dietary modifications during the night hours. Metabolites of amino acids, fatty acids, vitamins, and caffeine represent the major metabolomic changes found during the month of Ramadan.

## Data availability statement

This study was available at the NIH Common Fund’s National Metabolomics Data Repository (NMDR) website, the Metabolomics Workbench, https://www.metabolomicsworkbench.org where it has been assigned the Study ID ST002250. The data can be accessed directly via its Project DOI: http://dx.doi.org/10.21228/M8V71F.

## Ethics statement

The studies involving human participants were reviewed and approved by Research Ethics Committee, University of Sharjah. The patients/participants provided their written informed consent to participate in this study.

## Author contributions

MF and MM: conceptualization. MM, NS, and MS: methodology. NS and AG: software, formal analysis, and visualization. NS, MS, MM, and MF: validation. MF, MM, NS, and MS: investigation and resources. MF, MM, and NS: data curation. MF, MM, NS, and FZ: writing—original draft. AG, RH, and YB: writing—review and editing. MF and MM: supervision. MF: project administration, funding acquisition.
